# Pediatric thoracic spinal Ewing sarcoma/primitive neuroectodermal tumor: a case report and literature review

**DOI:** 10.3389/fped.2025.1603099

**Published:** 2025-12-16

**Authors:** Long Ma, Xiaodong Li, Fei Meng, Stanley Sau Ching Wong, Fengfeng Wang

**Affiliations:** 1 Department of Neurosurgery, Siping City Central People's Hospital, Siping City, Jilin Province, China; 2 Department of Anaesthesiology, School of Clinical Medicine, Li Ka Shing Faculty of Medicine, The University of Hong Kong, Hong Kong, Hong Kong SAR, China

**Keywords:** Ewing sarcoma/primitive neuroectodermal tumor (ES/PNET), thoracic spine, metastasis, prognosis, case report

## Abstract

The Ewing sarcoma/primitive neuroectodermal tumor (ES/PNET) is highly malignant neoplasms composed of undifferentiated small round cells with the ability to differentiate into various tissue types. We reported the case of an 11-year-old boy who presented with unsteady gait, progressive back pain, bilateral lower limb weakness (more pronounced in the left leg), and urinary retention. Magnetic resonance imaging (MRI) demonstrated a uniformly intense extradural lesion at the T3–5 vertebral level on the left posterolateral side of the spinal canal. The lesion measured 1.35 cm × 4.8 cm on pre-contrast MRI and 4.8 cm × 1.8 cm × 1.2 cm on post-contrast imaging, causing anterior and rightward displacement of the spinal cord with associated intramedullary signal changes and moderate post-contrast enhancement. Preoperative imaging suggested possible diagnoses of lymphoma or lipovascular tumor. However, postoperative histopathological examination confirmed the diagnosis of a small round cell malignant tumor consistent with an ES/PNET. The rapid progression to intramedullary metastasis and poor outcome emphasize the need for early diagnosis and more effective treatment strategies. Spinal ES/PNET is extremely rare, and this case highlights the clinical presentation, diagnostic challenges, and histopathological features of this aggressive tumor, which remain poorly reported in the literature.

## 1 Introduction

The Ewing sarcoma/primitive neuroectodermal tumor (ES/PNET) is a group of highly malignant neoplasms composed of undifferentiated small round cells with significant differentiation potential into various tissue types, including neuronal, gliomatous, and mesenchymal elements (1–3). These tumors originate from primitive neuroepithelium and are considered rare, particularly in the pediatric population (4).

The clinical presentation of ES/PNET often includes localized pain, which may be accompanied by neurological compression symptoms such as weakness, sensory deficits, or autonomic dysfunction, depending on the tumor's location (5). Primary spinal ES/PNET, in particular, is exceedingly rare, with only a few cases reported in the literature, making them a challenging diagnosis for clinicians (6).

This case report presents the clinical and radiological findings, as well as the histopathological diagnosis, of an 11-year-old boy with primary spinal ES/PNET. By documenting this rare case, we aim to enhance understanding and awareness of this uncommon condition among healthcare professionals.

*The informed consent was obtained from the patient's mother prior to the publication of this case report*.

## 2 Case report

A 11-year-old male patient was admitted to the Department of Neurosurgery at Siping Central People's Hospital on March 27, 2022, due to unsteady gait for 5 days and progressive weakness in both lower limbs that developed over the last 10 h (Table 1). The patient had been experiencing unsteady gait for 5 days prior to admission, but his symptoms were initially ignored by his mother, and no medical treatment was sought. On the day before admission, the unsteady gait persisted without improvement, prompting his mother to urgently seek medical attention.

**Table 1:** Follow up of the patient.

Timeline	Event description	Remarks
22/03/2022	First onset of unsteady gait	Initial symptom
27/03/2022	Admitted to hospital due to worsening bilateral lower limb weakness	Initial diagnosis, MRI revealed thoracic spinal mass
27/03/2022	Underwent thoracic spinal tumor resection	Intraoperative frozen pathology suggested malignancy
06/04/2022	Postoperative stabilization; transferred to oncology department	Initiated chemotherapy
10/05/2022	Second cycle of chemotherapy (regimen: epirubicin + cyclophosphamide + vindesine)	No local recurrence
30/05/2022	Admitted for bacteremia	Infectious complication
03/07/2022	Right hypochondrial pain with compression fracture	Suggested local recurrence
13/07/2022	Urinary and fecal incontinence	Neurological deterioration
18/07/2022	Worsening back pain	Advanced terminal stage
20/07/2022	Readmitted; MRI indicated intramedullary metastasis	Extensive metastasis
13/08/2022	Respiratory failure	Terminal state
14/09/2022	Right hypochondrial pain; Compression fracture; Urinary incontinence; Fecal incontinence	Loss of sensation in the lower body
30/10/2022	Numbness in both upper limbs	Involvement of the high cervical spinal cord
26/11/2022	Quadriplegia	High-level paraplegia due to spinal cord injury
16/12/2022	Development of aspiration pneumonia	Involvement of the fifth cervical spinal cord leading to weakened diaphragmatic breathing and subsequent aspiration pneumonia
13/01/2023	Death	Tumor invasion of upper cervical spinal cord leading to respiratory failure

### 2.1 Clinical presentation

A cranial magnetic resonance imaging (MRI) scan without contrast and diffusion-weighted imaging (DWI) suggested the possibility of a right temporal arachnoid cyst. However, the patient was not hospitalized and was sent home to rest. Ten hours before admission, the patient developed back pain, bilateral lower limb weakness (more severe in the left leg), and urinary retention. As a result, the family returned to the outpatient clinic for further evaluation. The patient had no significant medical history or family history of genetic diseases.

Upon admission, a physical examination was conducted. The patient's vital signs were as follows: temperature, 36.4°C; pulse, 73 beats per minute; respiration, 18 breaths per minute; and blood pressure, 119/74 mmHg. The patient was conscious, with hyperesthesia below the left third rib. Muscle strength was grade 5 in both upper limbs, grade 3 in the left lower limb, and grade 4 in the right lower limb. Muscle tone was normal in all limbs, and bilateral Babinski signs were absent.

### 2.2 Radiological findings

Blood test results were unremarkable. A three-dimensional computed tomography (3D CT) scan of the thoracic spine revealed no abnormalities in the intervertebral foramina or in the thoracic vertebral bones. On March 27, 2022, a 1T MRI scan of the entire spine identified an abnormal extradural signal at the T3–5 vertebral level, situated predominantly on the left and posterior sides of the spinal canal. The lesion appeared as a strip-like structure with long T1 and long T2 signal characteristics, uniformly intense, measuring approximately 1.35 cm × 4.8 cm. The spinal cord was significantly compressed and displaced anteriorly to the right, with uneven signals noted in the corresponding spinal cord segment.

**Figure 1:**
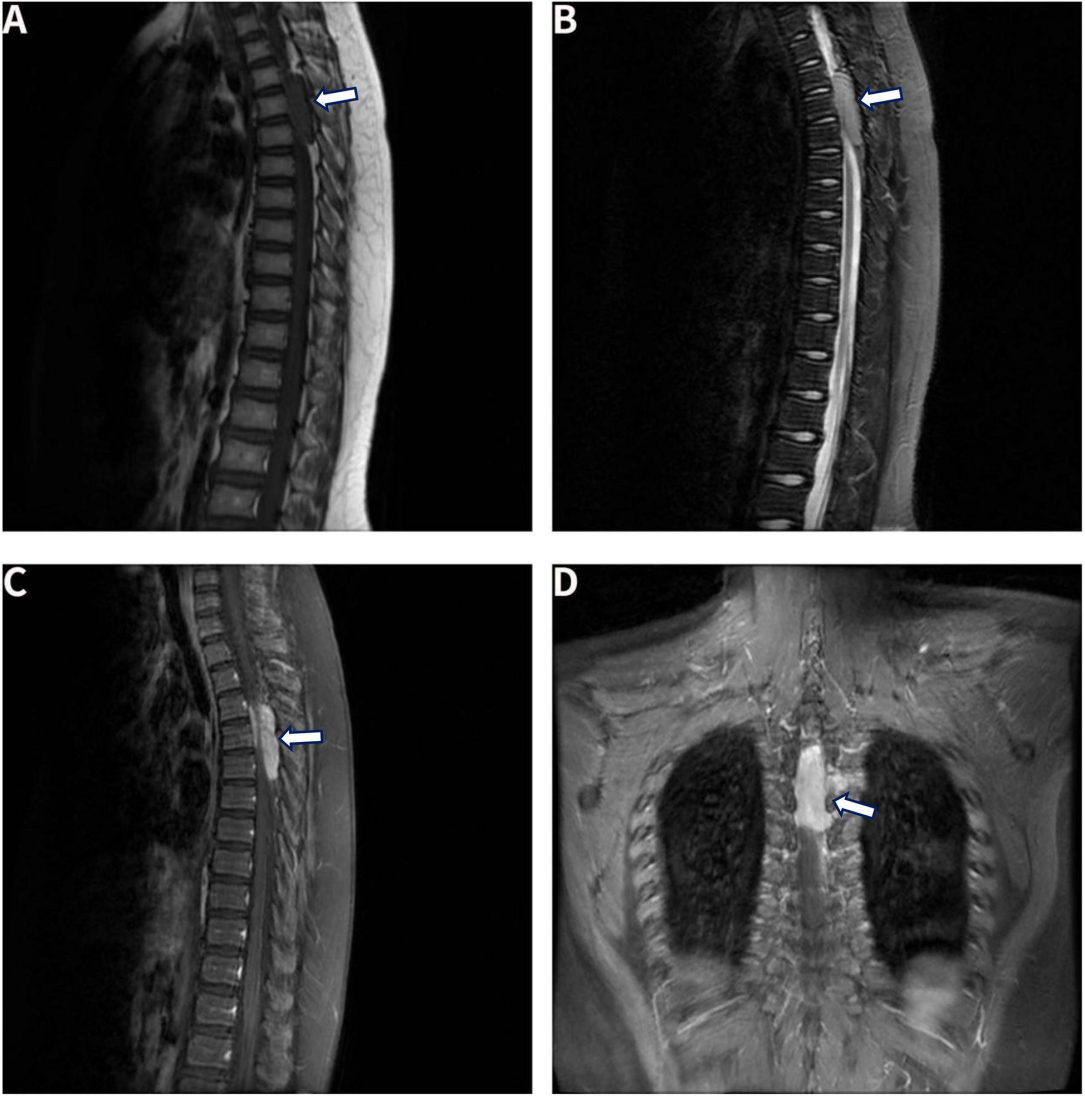
MRI scan of the thoracic spine showed an oval-shaped, long T1 and long T2 signal shadow within the extradural space at the T3–5 level. **(A)** T1-weighted image. **(B)** T2-weighted image. **(C)** and **(D)** Sagittal and coronal enhanced MRI images. Arrows indicate the locations of the lesions.

A follow-up enhanced MRI scan of the thoracic spine showed an oval-shaped, long T1 and long T2 signal shadow within the extradural space at the T3–5 level. The enhancement was moderate after contrast injection, with the lesion measuring approximately 4.8 × 1.8 × 1.2 cm. The adjacent dura mater also exhibited enhancement. Importantly, the spinal cord remained compressed and displaced to the right, but no abnormal signal enhancement was observed in the spinal cord itself at the corresponding level. The left side of the lesion extended into the left intervertebral foramen, forming a “dumbbell” shape. A strip-like area of uniform enhancement was also noted in the T5 transverse process and the spinous muscle (Figure 1). The preliminary preoperative diagnosis included lymphoma or lipovascular tumor as potential differential diagnoses.

### 2.3 Surgical procedure and intraoperative findings

**Figure 2:**
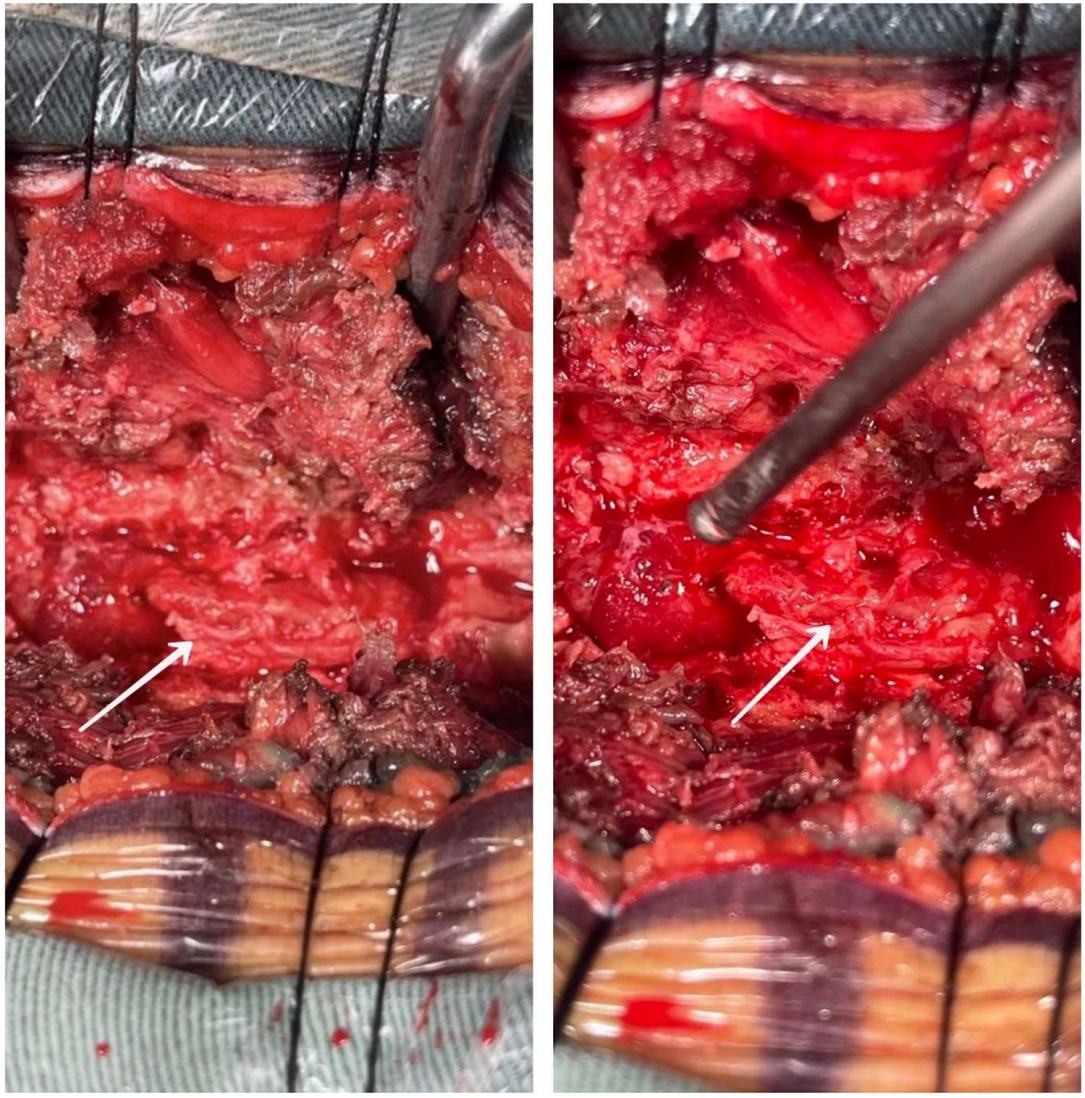
The resection and imaging of the intraspinal lesion performed under general anesthesia. The tumor was characterized by a bright red appearance, a firm texture, and a rich blood supply.

The treatment plan was to perform a posterior midline approach for exploration and resection of the intraspinal lesion under general anesthesia. Surgical findings revealed a mass located between the T3–5 spinous processes, which was bright red in color, firm, and highly vascularized (Figure 2). The mass was removed and sent for rapid intraoperative pathology.

The intraoperative pathology report suggested the possibility of malignant lymphoma. During the procedure, the remaining tumor mass was removed as completely as possible under gross and microscopic examination. Notably, local fine osteolytic destruction, which was not visible on the MRI plain film, was still observed. However, the portion of the dura mater attached to the tumor was not resected to avoid unnecessary complications.

### 2.4 Histopathological and immunohistochemical findings

**Figure 3:**
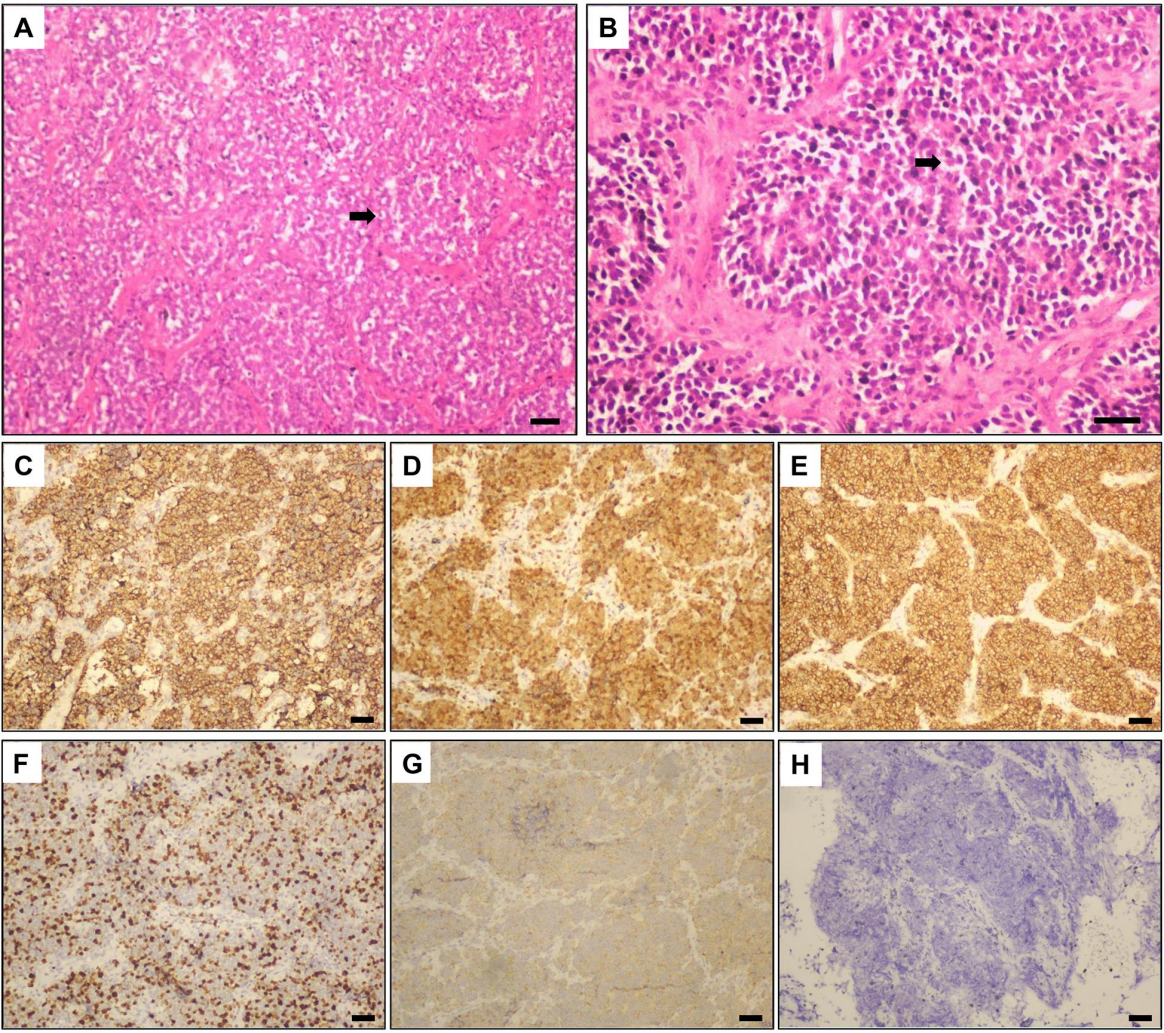
Histopathological and immunohistochemical images. Hematoxylin and Eosin (H&E)-stained histopathological images at **(A)** 100x & **(B)** 200× magnification: A large number of small round or spindle-shaped primitive cells were densely packed, with hyperchromatic nuclei, scant cytoplasm, and focal rosette formations, consistent with a small round cell malignant tumor. Immunohistochemistry (100× magnification) for **(C)** Diffuse positivity for CD99; **(D)** Positive staining for CyclinD1; **(E)** Positive expression of CD56; **(F)** KI67 positivity (80%); **(G)** Positive staining for Synaptophysin; and **(H)** Negative staining controls for immunohistochemistry in this patient. Scale bars = 50 μm. Arrows indicate rosette-like cell clusters.

Postoperative histopathological examination revealed a small round cell malignant tumor in the spinal canal, consistent with an ES/PNET (Figures 3A,B). Immunohistochemical staining further supported this diagnosis, with the following results: CD99 diffuse (+), CyclinD1 (+), CD56 (+), and KI-67 index of 80% (+) (Figures 3C–F). Figure 3G demonstrates positive synaptophysin expression, whereas Figure 3H illustrates negative expression in the patient's pathological tissue by immunohistochemistry. These findings confirmed the diagnosis of ES/PNET as the definitive pathology of the lesion.

Under light microscopy, the tumor was composed of uniformly small, round, blue cells with high nuclear-to-cytoplasmic (N/C) ratios, delicate chromatin, and indistinct nucleoli. Mitotic figures were numerous and easily identifiable, with an estimated count exceeding 50 mitoses per 10 high-power fields (HPF), indicative of a highly proliferative tumor. Areas of coagulative necrosis were identified within the tumor. These necrotic regions appeared as large geographic or map-like zones composed of eosinophilic cellular debris, often with collapsed architecture and pyknotic or karyorrhectic nuclear fragments. Homer-Wright rosettes were visibly present within the tumor (Figures 3A,B).

### 2.5 Postoperative status

**Figure 4:**
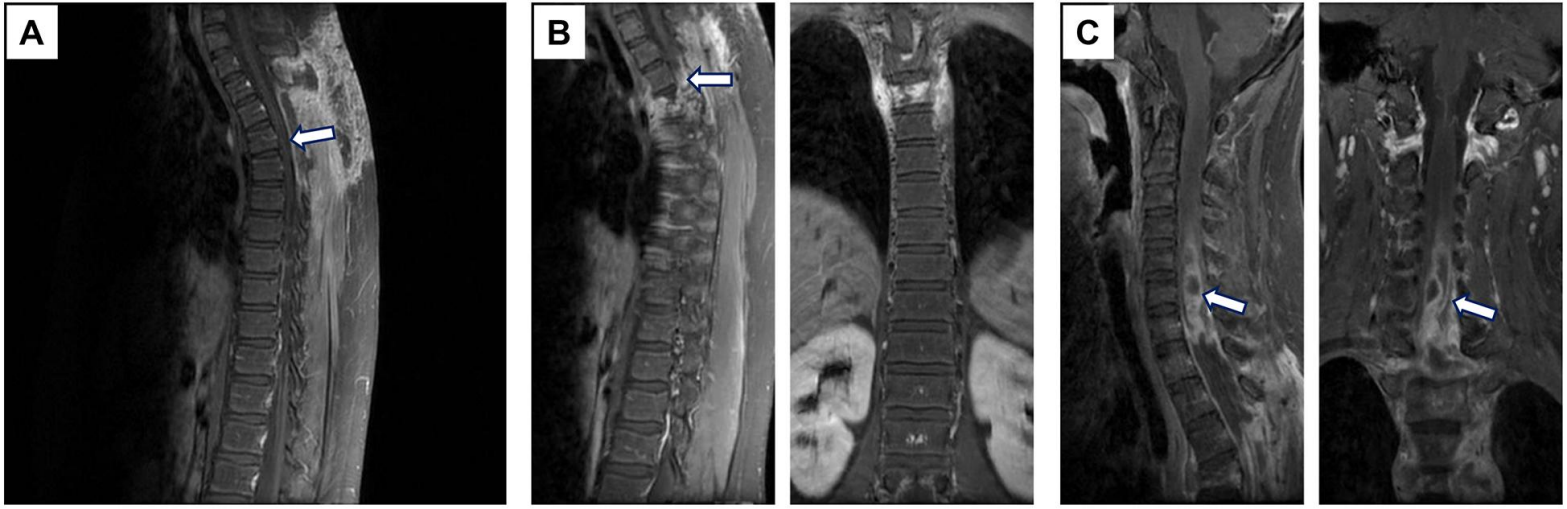
MRI imaging after treatment. **(A)** After the second cycle of chemotherapy, MRI showed no detectable signs of recurrence. **(B)** Due to poor response to chemotherapy, radiotherapy was initiated. However, tumor recurrence was observed, along with vertebral body fracture and spinal cord compression. **(C)** Evidence of intramedullary metastasis was noted on subsequent imaging. Arrows indicate the locations of the lesions.

After surgery, the patient regained mobility and was transferred to the oncology department on April 6, 2022, for chemotherapy after his condition stabilized. The chemotherapy regimen included epirubicin, cyclophosphamide, and vincristine (7). A second round of chemotherapy was administered on May 10, 2022, in the oncology department, with no signs of local recurrence observed at that time (Figure 4A).

However, the patient experienced complications postoperatively. On May 30, 2022, he was hospitalized due to bacteremia. On July 3, 2022, he was rehospitalized for one day due to discomfort in the right hypochondrium, during which imaging revealed a compression fracture, suggesting possible tumor recurrence (Figure 4B). Due to the poor response to chemotherapy, radiotherapy was initiated.

The patient's condition continued to deteriorate. On July 13, 2022, he experienced difficulty with defecation and urination. By July 18, 2022, his back pain had worsened significantly, leading to readmission on July 20, 2022. An MRI review on August 13, 2022, indicated intramedullary metastasis (Figure 4C).

The patient's neurological status progressively declined. On September 14, 2022, upon readmission, he exhibited loss of pain and touch sensation below the nipple level, with muscle strength graded as I in both lower limbs, III in the left upper limb, and mild edema in both lower limbs. On October 30, 2022, numbness developed in the upper limbs, followed by worsening limb dysfunction on November 26, 2022.

In December 2022, the patient developed hypostatic pneumonia, which progressed to respiratory failure on January 13, 2023. The patient ultimately passed away due to tumor invasion of the high cervical spinal cord, leading to respiratory failure.

## 3 Discussion

The Ewing sarcoma/primitive neuroectodermal tumor is rare, aggressive malignancies derived from primitive neuroepithelium, predominantly affecting adolescents. These tumors are clinically challenging due to their rapid growth, high tendency for local invasion, and early metastasis, which often result in poor outcomes, with survival rates typically limited to less than five years. In this case, the preoperative cranial MRI showed no abnormalities, excluding metastatic disease and supporting the diagnosis of a primary spinal ES/PNET.

Spinal ES/PNET can arise in various locations within the spinal canal, including the extradural space, subdural extramedullary region, intramedullary space, or outside the spinal canal at any spinal segment (8, 9). Lesions in the extradural space lack specific imaging features, making them difficult to differentiate from other spinal tumors, such as Ewing sarcoma (8). On MRI, both can appear as low, iso-, or hyperintense signals on T1-weighted imaging (T1WI), low or hyperintense signals on T2-weighted imaging (T2WI), and exhibit uneven enhancement. Hemorrhage or necrosis, which are sometimes present, may further complicate differentiation based on imaging alone. MRI remains the most effective modality for evaluating tumor extension and its relationship to surrounding structures, although definitive diagnosis relies on histopathological examination (10).

The diagnosis of spinal ES/PNET is primarily based on pathological analysis, with immunohistochemical studies playing a crucial role in distinguishing it from other conditions, such as lymphoma, eosinophilic granuloma, spinal Rosai-Dorfman disease, and vascular lipoma (8). Grossly, the tumor often appears as a purple-red or gray-white mass, with a soft or firm texture and abundant blood supply. Microscopically, spinal ES/PNET is characterized by densely packed small round or spindle-shaped primitive cells with hyperchromatic nuclei, scant cytoplasm, and occasional rosette formations (11).

This patient's immunohistochemical results revealed the following: CD99 diffuse (+), CyclinD1 (+), CD56 (+), and KI-67 index of 80% (+). Immunohistochemistry is particularly useful for distinguishing PNET subtypes. Peripheral PNETs (pPNETs) typically express CD99, while central PNETs (cPNETs) do not. Since the lesion in this patient was CD99-positive, it was consistent with a diagnosis of pPNET. Additionally, genetic studies have shown that over 90% of pPNETs exhibit the characteristic t(11;22)(q24;q12) chromosomal translocation, which is not observed in cPNETs (12, 13). While BRAF mutations are rarely seen in PNETs (approximately 3% of cases), their presence does not correlate with prognosis (8, 14).

A principal limitation of this case is the inability to perform molecular testing for the EWSR1-FLI1 gene fusion, which is the current diagnostic standard for definitive diagnosis of Ewing sarcoma (2, 3, 15). This limitation was due to constraints in our hospital's molecular pathology laboratory resources and testing capabilities at the time of diagnosis. Another limitation is the lack of an extended immunohistochemical (IHC) panel in the initial diagnostic workup, which could have further refined the differential diagnosis of small round blue cell tumors (SRBCTs) (2, 3). Specifically, immunohistochemical markers such as Desmin (a marker of rhabdomyoblastic differentiation), Myogenin (specific for skeletal muscle lineage), INI1 (loss of expression observed in rhabdoid tumors and certain SRBCTs), FLI-1 (a sensitive marker for Ewing sarcoma), and NB84 (a marker for neuroblastoma) were not assessed. This was primarily due to institutional IHC protocol limitations and sample-related considerations at the time. Consequently, the diagnosis was established based on histopathological findings, immunohistochemical staining (including strong and diffuse CD99 positivity), and clinical correlation, with particular attention to the patient's age and the anatomical location of the lesion.

Modern proton therapy represents a significant advancement in the treatment of spinal ES/PNET. This highly precise modality minimizes radiation exposure to surrounding normal tissues while delivering high doses to the tumor, resulting in improved tumor control and better quality of life for patients. Proton therapy has shown particular promise in the treatment of pediatric malignancies, head and neck cancers, chordomas, and cervical cancers (16). International case reports have described patients with dorsal ES/PNET who, despite local recurrence, remained disease-free for up to 10 years after receiving extensive radiotherapy.

In this case, the patient's clinical course points out the aggressive nature of spinal ES/PNET. Postoperatively, the patient initially showed improvement and was started on chemotherapy. However, the tumor recurred, as evidenced by imaging findings of intramedullary metastasis and later complications, including hypostatic pneumonia, respiratory failure, and eventual death. These outcomes highlight the challenges associated with managing spinal ES/PNET in adolescents.

ES/PNET has been reported in the literature in a number of previous cases (17, 18). The standard treatment for ES/PNET combines systemic and local therapies, including multiagent chemotherapy, surgical resection, and radiotherapy, to control the primary tumor and address potential metastases (19, 20). Despite improvements in conventional therapy, survival outcomes for spinal ES/PNET remain poor. Recent studies and case reports have investigated novel approaches such as targeted agents against EWSR1-FLI1, the PI3K-Akt-mTOR pathway, and CD99, as well as immunotherapies including checkpoint inhibitors and CAR T-cell therapy, which have shown early promise in refractory or relapsed cases (21–24). Prognosis is shaped by several factors consistently reported in both series and case reports. Metastasis at presentation is the most significant adverse predictor, with gross total resection linked to better local control and survival than subtotal resection or biopsy alone (19). Younger patients generally have more favorable outcomes than adults (25, 26), and while the EWSR1-FLI1 fusion is diagnostic, additional genetic alterations may offer further prognostic stratification (21, 27, 28).

This case presents several novel aspects compared to previously reported cases of spinal ES/PNET. First, the absence of cranial abnormalities on preoperative imaging excluded metastatic disease and supported the diagnosis of a primary spinal ES/PNET, which is relatively rare. Additionally, the lesion's unique “dumbbell” shape with extension into the left intervertebral foramen and its progression to intramedullary metastasis postoperatively highlight the aggressive and atypical behavior of this tumor. These findings are less commonly reported and emphasize the challenges in diagnosing and managing spinal ES/PNET with such presentations. Another notable difference is the development of multiple complications postoperatively, including bacteremia, compression fracture, and hypostatic pneumonia, which ultimately contributed to the patient's poor outcome. These complications emphasize the need for comprehensive perioperative care and close monitoring in patients with spinal ES/PNET. Clinically, this case highlights the importance of early diagnosis and the limitations of current treatment modalities, including surgery, chemotherapy, and radiotherapy, in achieving long-term control of this aggressive tumor. It also reveals the potential role of advanced imaging techniques and novel therapies, such as proton therapy or gene therapy, in improving outcomes for patients with spinal ES/PNET. Overall, this case adds valuable insights into the clinical presentation, diagnostic challenges, and management strategies for spinal ES/PNET, particularly in pediatric and adolescent populations.

Spinal ES/PNET typically present as extradural masses with CT findings of medium-density soft tissue and MRI findings of low T1WI and iso- or hypointense T2WI signals, often with cystic changes (29). The final diagnosis, however, is based on histopathological and immunohistochemical findings. In adolescents presenting with a single extradural spinal mass accompanied by extraspinal components, irregular CT density, or MRI signal with significant uneven enhancement, ES/PNET should be included in the differential diagnosis (30, 31).

Despite advances in multimodal treatment, including surgery, radiation therapy, and chemotherapy, the prognosis for spinal ES/PNET remains poor, with short survival times (32). Molecular targeted therapy may hold promise as a future treatment option, potentially offering more effective management of this aggressive disease (19).

## Data Availability

The original contributions presented in the study are included in the article/Supplementary Material, further inquiries can be directed to the corresponding author.
